# Investigating patient nonattendance at healthcare appointments in Türkiye: data from a state hospital and an oral and dental health center

**DOI:** 10.55730/1300-0144.6086

**Published:** 2025-07-26

**Authors:** Eyüp SARI, Ali Niyazi KURTCEBE, Cihan DÖĞER, Recep AYDIN

**Affiliations:** Ankara Provincial Health Directorate, Ankara, Turkiye

**Keywords:** Appointment nonattendance, healthcare, demographics, patient compliance, resource utilization

## Abstract

**Background/aim:**

To determine the frequency and reasons for appointment nonattendance in a state hospital and a dental health center in a densely populated district of Türkiye, and to identify related factors to guide preventive measures.

**Materials and methods:**

This retrospective cross-sectional study included patients who scheduled appointments at Ankara Etimesgut State Hospital and Etimesgut Oral and Dental Health Center through the Centralized Patient Appointment System (Merkezi Hekim Randevu Sistemi; MHRS) in February 2024. Nonattendees were contacted in March 2024 to determine reasons for nonattendance.

**Results:**

A total of 30,552 appointments were analyzed (1978 dental, 28,574 hospital). The overall nonattendance rate was 9.76%, with significantly higher rates in the dental center (23.8%) compared to the state hospital (2.2%) (p < 0.001). Males had higher nonattendance rates than females (4.04% versus 3.36%, p = 0.002), and the 19–45 age group showed the highest nonattendance (5.63%, p < 0.001). The most common reasons were forgetfulness (20.7%), scheduling conflicts (16.5%), and illness (13.8%).

**Conclusion:**

Appointment nonattendance is influenced by demographics and timing. Comprehensive reminder systems and flexible scheduling policies may effectively reduce nonattendance rates, particularly among young adults, thereby improving patient adherence and healthcare resource utilization.

## 1. Introduction

Outpatient clinics, commonly known as polyclinics in Türkiye, play a crucial role in the healthcare system by being the first point of contact in the hospital and providing a wide range of medical services that do not necessitate hospitalization. These clinics offer specialist examinations in the respective departments, the possibility for consultation and referrals, diagnostic tests, and minor procedures [1]. While previous Turkish studies have examined national MHRS data patterns, this study uniquely investigates appointment adherence across two distinct healthcare settings within a single district, allowing for direct comparison between general medical and dental care attendance behaviors.

In Türkiye, appointments for polyclinic visits are managed with a centralized system that serves the whole country (Merkezi Hekim Randevu Sistemi; MHRS), translated as the Central Physician Appointment System. Internationally, appointment systems vary widely, with centralized systems like MHRS comparable to the UK’s National Health Service (NHS) e-Referral Service and Canada’s centralized triage models. The MHRS seeks to optimize the booking of appointments with specialist physicians by allowing appointment search throughout multiple institutions, thereby reducing waiting times and improving access to healthcare services [2]. Despite offering rescheduling and cancellation opportunities within the same system, patients not showing up for their scheduled appointments has become a considerable problem, impacting both the efficiency of healthcare services and the well-being of other patients in need of timely medical care. These issues have been examined by multiple studies [3–7] that have described the frequency and reasons for nonattendance at scheduled appointments. Among the reasons for nonattendance, various factors have been reported to take priority, including age, sex, the season of the appointment, and distance to the hospital. International studies report appointment nonattendance rates ranging from 5%–30% depending on healthcare systems and cultural contexts, with similar demographic patterns observed across developed nations. However, the impact of centralized appointment systems like Türkiye’s MHRS on attendance patterns remains understudied.

The effective and efficient work of healthcare professionals is vital to ensuring public access to healthcare services and increasing satisfaction. Determining the frequency and reasons for nonattendance at healthcare appointments can inform the development of measures and policies to address this widespread problem. We therefore sought to examine the frequency of nonattendance at healthcare appointments and investigate factors associated with this phenomenon in a state hospital and an oral and dental health center located in a densely populated district in Ankara, Türkiye.

## 2. Materials and methods

In this cross-sectional study, all patients who made an appointment during February 2024 through the MHRS system for examination or treatment at Etimesgut State Hospital and Etimesgut Oral and Dental Health Center in Ankara, Türkiye were examined. Ethical approval was obtained from the Ankara Bilkent City Hospital Clinical Research Ethics Committee (approval number: TABED 2-24-692, date: 27 November 2024) in accordance with the principles of the Declaration of Helsinki.

All patients who scheduled appointments at the two health institutions via MHRS were eligible for inclusion. Inclusion criteria comprised all patients aged 0–99 years who scheduled appointments via MHRS for routine examination or treatment. Exclusion criteria were: (1) missing demographic data in hospital records, (2) unreachable phone numbers after three call attempts, (3) inability to communicate in Turkish, and (4) appointments for emergency procedures. The data for the cases were retrospectively accessed from the Turkish Ministry of Health registration system. Within the scope of the study, sex, age, appointment specialty, appointment date and time, and appointment attendance status were recorded. Patients who canceled appointments ≥ 24 h in advance through MHRS were excluded from the nonattendance group, as were those who rescheduled within the system.

Nonattendees were contacted via telephone between 1–15 March 2024, using a standardized script. Each patient (or parent for those < 18 years) was asked: “What was the primary reason you did not attend your scheduled appointment on [date]?” Verbal consent was obtained before questioning. Up to three call attempts were made at different times of day. Response rate was 73.2% (1096/1497 contacted). February 2024 was selected as it represents a typical month with mixed seasonal factors, including the end of the semester break (1–9 February) allowing examination of appointment attendance across different social contexts within a single month. We acknowledge that February includes a semester break period (1–9 February), which may have influenced attendance patterns and has been discussed as a potential confounding factor. Those with missing data in hospital records, those who could not be reached by phone after missing their appointments, or those who could not communicate were excluded from the study.

According to the study exclusion criteria, the study was conducted with 27,949 state hospital patients who attended appointments and 625 who did not attend, plus 1507 dental center patients who attended and 471 who did not attend.

Sample size was determined by the total eligible appointments during the study period. Post hoc power analysis indicated > 80% power to detect clinically meaningful differences (≥ 2%) in attendance rates between demographic groups. Phone contact success rate was 73.2%, with nonresponse primarily due to disconnected numbers (15.3%) and failure to answer after three attempts (11.5%).

### 2.1. Statistical analysis

Analyses were performed with SPSS version 26.0 (IBM Corp., Armonk, NY, USA). Categorical variables were expressed as number (%) values and relationships were analyzed with chi-square test. The compatibility of numerical data with the normal distribution was evaluated using the Kolmogorov–Smirnov test. Continuous numerical data were summarized with mean ± standard deviation values. The significance limit was accepted as p < 0.05.

## 3. Results

In this study, which included 1978 appointments to the dental center and 28,574 appointments to the state hospital during February 2024, 33.6% of the patients were male and the mean age was 40.94 ± 21.17 years. The most common departments for which appointments were made were internal medicine (15.9%), pediatrics (11.4%), and gynecology and obstetrics (9.9%) (Table 1).

The most common reasons for not attending appointments were forgetfulness (20.7%), scheduling conflicts (16.5%), illness (13.8%), and failure to arrive on time (11.0%) (Table 2).

Compared to women, the frequency of nonattendance was significantly higher in men (p = 0.002). Patients between the ages of 19–45 years were significantly more likely to not attend appointments compared to other age groups (p < 0.001). There was a significant difference in nonattendance rates between departments (p < 0.001). The departments with the lowest appointment attendance rates were psychiatry (6.0%), neurology (4.2%), and infectious diseases (4.1%), respectively (Table 3).

Departments were categorized by nonattendance rates: high nonattendance specialties (psychiatry [6.0%], neurology [4.2%], infectious diseases [4.1%]), moderate nonattendance specialties (dermatology [3.9%], neurosurgery [3.4%], ENT [3.1%]), and low nonattendance specialties (pediatrics [0.4%], internal medicine [1.2%], cardiology [1.7%]).

**Figure 1:**
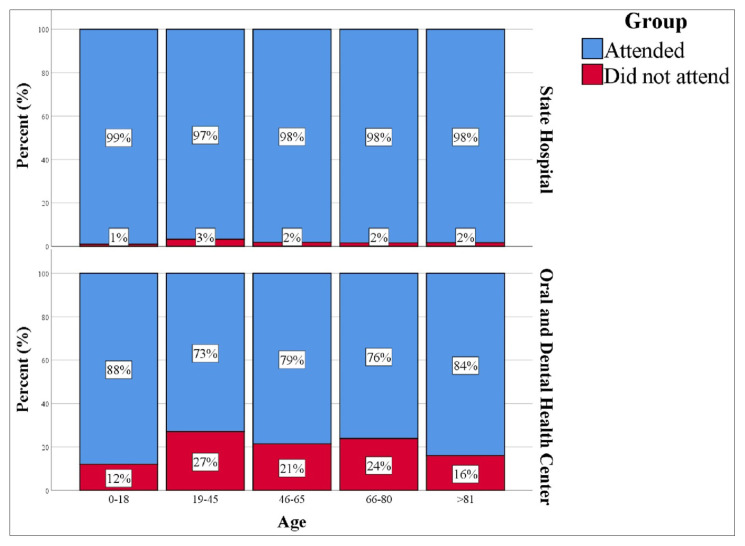
Frequency of nonattendance at hospital appointments by age group.

**Figure 2:**
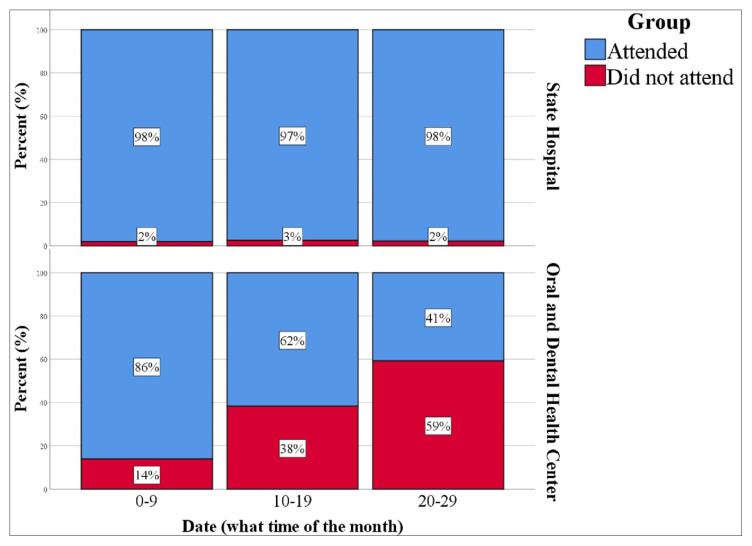
Frequency of nonattendance at hospital appointments by center and appointment date.

When the hospital appointments were analyzed, no difference was found between the sexes in terms of nonattendance (p = 0.165). Patients aged 19–45 years did not attend their appointments significantly more frequently than other age groups (p < 0.001, Figure 1). Compared to those with appointments between 1–9 February, those with appointments between 10–19 February had a significantly higher frequency of nonattendance (p = 0.005, Figure 2). In addition, there was a significant correlation between specialty and nonattendance (p < 0.001) (Table 4).

For the dental center, it was determined that those in the 0–18 age group attended significantly fewer appointments than those in other age groups (p < 0.001, Figure 1). In addition, the highest frequency of nonattendance was observed between 20–29 February (59.2%), followed by 10–19 February (38.2%), while the lowest was between 1–9 February (13.8%) (p < 0.001, Figure 2; Table 5).

## 4. Discussion

This study reports the frequency of nonattendance at healthcare appointments and the factors contributing to nonattendance in a high-demand healthcare setting in Türkiye. The findings indicate that nonattendance was more prevalent among males and young adults, and that it varied by medical specialty. Key reasons included forgetfulness, unexpected obligations, and illness, largely reflecting individual challenges in patient adherence. These results align with previous studies that identified similar trends but also reveal unique patterns observed in our population.

In our setting, approximately one out of every 10 patients did not show up for their hospital appointment. This frequency was lower than some similar studies conducted in Türkiye in previous years. In a study evaluating 2-years of MHRS records from a tertiary hospital in Türkiye, it was found that 22% of patients who made an appointment did not attend [8]. In another comprehensive study conducted in Türkiye using nationwide MHRS data, it was reported that patients did not attend 22.2% of hospital appointments [9]. Zorlu and Kavurmacı investigated the frequency and reasons for nonattendance at thoracic surgery outpatient clinic appointments in Türkiye and found that 18.8% of patients did not attend their appointments and 8.6% received incorrect appointments [10]. Although similar frequencies have been reported previously, taken together, it appears that nonattendance rates in our study are relatively low compared to most prior studies. This may be related to the fact that this study was conducted in a developed urban center. The relatively low nonattendance rates in our study compared to earlier Turkish research may reflect recent MHRS system improvements, including automated SMS reminders introduced in 2023 and the 15-day rebooking restriction policy.

In a recent review including studies examining the frequency and reasons for nonattendance at hospital appointments in many different countries, the nonattendance rate was reported to be 5%–30%, depending on the sociodemographic and cultural structure of the society in which the study was conducted [11]. For instance, studies from Australia and Germany report rates around 15%–20% [5,17], while some US-based research has found rates up to 30% [11]. Compared to these figures, the nonattendance rate observed in our setting appears relatively low. While international studies report wide variation in nonattendance rates across different healthcare systems and cultural contexts, direct comparisons are challenging due to differences in appointment systems, patient populations, and measurement methods. Our findings contribute to the growing body of evidence on appointment adherence in centralized healthcare systems. The MHRS system’s built-in attendance enforcement mechanisms likely contributed to our relatively low nonattendance rates. Patients who miss appointments without 24 h notice face a 15-day restriction on booking new appointments in the same specialty, creating a strong incentive for attendance or proper cancellation.

In our study, one out of every five patients who did not attend a hospital appointment reported that they missed the appointment because they forgot. This excuse ranked first among the reasons for nonattendance. In many previous studies, forgetting the appointment was reported as the most common reason for nonattendance with a similar frequency [5,12–21]. In a systematic review in which Werner et al. examined the importance of economic interventions to reduce nonattendance at hospital appointments, especially to prevent forgetting, they found that the most frequently used and successful methods were sending text messages as appointment reminders, making voice calls, or sending e-mails [22]. Similarly, in Türkiye, notifications are sent to the phone numbers of the patients registered in the system. Additionally, those who do not cancel their appointments at least 1 day in advance are restricted from making new appointments for the same specialty within the next 15 days. This intervention should also be taken into account when interpreting the results of our study, especially the frequency of nonattendance due to the “forgetting” factor. It should be noted that self-reported reasons for nonattendance may be subject to social desirability bias, with patients potentially underreporting preventable reasons such as forgetting or lack of interest in healthcare.

In our study, the frequency of nonattendance at appointments was significantly higher in men compared to women. We hypothesize that higher nonattendance among males may be related to greater workforce participation rates in Türkiye [23], though this relationship requires further investigation with employment-specific data. This significant relationship found in our study has been described in various studies. Some researchers found that men [3–6,24–26] missed their appointments more frequently; however, although few in number, there are studies that have found that women [7,27] missed their appointments more frequently. Furthermore, other studies have reported no significant difference between the sexes regarding nonattendence at healthcare appointments [28–34]. A holistic review of the literature largely shows that there are fewer studies suggesting that women demonstrate lower attendance rates. This could be related to the fact that men are more reluctant to seek care due to nonemergency health problems [5], which might also be true for our study.

The highest appointment attendance levels were found in the 0–18 age group, while the lowest was in the 19–45 age group. Similar to our interpretation regarding sex, we think that one of the main reasons for this situation is that individuals in the 19–45 age group are active labor force participants. Since they are actively working, they may have had difficulty taking time off, may not have found time to attend the appointment, or may have forgotten their appointment due to their busy schedule. Another reason could be that young adults are less likely to have serious and chronic diseases. In addition, children usually attend hospital appointments with their parents and their parents demonstrate a greater level of care about this responsibility, which may be related to the higher frequency of hospital appointments in this age group. Similar to our study, some previous studies found that the frequency of nonattendance at hospital admissions was higher in younger adults [3,4,6,24,26–28,34–37]. Notably, in some rare studies, nonattendance was found to be more frequent in older individuals [33], while others were unable to find any significant relationships between age and attendance at hospital appointments [5,30,31]. As a result, the finding of our study can be interpreted as compatible with the literature. Young adults have low rates of hospital appointment attendance.

We observed that the frequency of nonattendance at appointments in both the state hospital and the dental center was lowest in the first 9 days of the month and increased throughout the rest of the month. This may be related to the fact that schools in Türkiye are on semester break at the beginning of February, providing more opportunities for making and keeping appointments. The end of the school vacation may have been a primary reason preventing attendance at hospital appointments for both university students and parents with school-age children.

The frequency of nonattendance at appointments was also found to differ significantly according to departments. This may be related to the difference in the treatment needs, severity of the disease and recovery time of the patients who scheduled appointments in different departments.

This study has certain limitations that should be acknowledged. The research design is retrospective and relies on data from a specific time frame (February 2024), which may limit the generalizability of the findings to other periods or settings. We did not examine different parameters that may affect nonattendance at hospital appointments such as marital status [3,35,38–42], education level [3,7,14,27,31,43], distance between the hospital and home [7,21,38–40], referral by primary care [5,24,26,27,30,32], and receiving an incorrect appointment [10]. Additionally, the study included only one hospital and one dental healthcare center in a single urban region in Ankara, Türkiye. Findings may not generalize beyond the urban district studied. Different settings and institutions are bound to impact the nonattendance frequencies and the reasons reported by individuals. Lack of multivariate analysis limits ability to identify independent predictors. Telephone-based follow-up for patients who did not attend appointments may also have introduced bias, as responses could be influenced by recall errors or nonresponse from certain groups. Only 25.8% of nonattendees responded our telephone calls, raising concern for response bias. Future studies encompassing multiple institutions and larger populations over extended periods of time are necessary to corroborate the present findings and understand the factors impacting this nationwide problem.

These findings suggest several policy interventions: (1) targeted SMS reminders for males aged 19–45, (2) flexible evening scheduling for working-age adults, (3) enhanced reminder systems for dental appointments, and (4) educational campaigns on the importance of timely cancellation.

Appointment nonattendance is a significant issue in healthcare services, with notable prevalence among specific demographic groups. In particular, males, young adults, and those who made appointments with certain specialties showed higher rates of nonattendance. The findings revealed that forgetfulness, scheduling conflicts, and illness were the primary reasons for missing appointments. Based on prior literature and the changes observed in this study, we believe that implementing comprehensive reminder systems, allowing for flexible scheduling policies, and providing targeted educational and awareness programs will be essential to enhance patient compliance with healthcare appointments. These strategies and interventions can positively influence patient behavior, improve efficiency, and minimize inefficient allocation of resources.

## Figures and Tables

**Table 1:** Distribution of sex, age, and appointment characteristics.

Variables	n	%
**Sex**		
Male	10,268	33.61
Female	20,284	66.39
**Age group (years)**		
0–18	5355	17.53
19–45	11,624	38.05
46–65	9573	31.33
66–80	3501	11.46
≥81	499	1.63
**Appointment center**		
Dental Health Center (DHC)	1978	6.47
State Hospital (SH)	28,574	93.53
**Department of appointment**		
Internal medicine	4864	15.92
Pediatrics	3468	11.35
Obstetrics and gynecology	3010	9.85
Orthopedics and traumatology	2467	8.07
General surgery	2092	6.85
Ophthalmology	2023	6.62
Oral and dental health	1978	6.47
Physical medicine and rehabilitation	1654	5.41
Otolaryngology (ENT)	1419	4.64
Neurology	1325	4.34
Urology	1153	3.77
Pulmonary diseases	1135	3.71
Dermatology and venereology	1071	3.51
Cardiology	1055	3.45
Psychiatry	697	2.28
Neurosurgery	615	2.01
Infectious diseases and clinical microbiology	317	1.04
Other departments	209	0.68
**Appointment date**		
February 1–9	11,213	36.70
February 10–19	9489	31.06
February 20–29	9850	32.24
**Appointment time**		
Morning	16963	55.52
Afternoon	13589	44.48

**Table 2:** Distribution of appointment attendance status and reasons for nonattendance.

Variables	n	%
**Attendance status**		
Attended	29,456	96.41
Did Not Attend	1096	3.59
**Reason for nonattendance**		
Forgot	227	20.71
Had a scheduling conflicts or other engagement	181	16.51
Due to illness	151	13.78
Could not arrive on time	121	11.04
Could not obtain leave	118	10.77
Appointment cancellation issue	75	6.84
System error	63	5.75
Out of town	61	5.57
Family issue	33	3.01
Other healthcare facility	24	2.19
Physician-related	18	1.64
Social security issue	10	0.91
Transportation issue	9	0.82
Documentation problem	5	0.46

**Table 3:** Factors associated with patients’ attendance at appointments.

	Attended (n = 29,456)		Did Not Attend (n = 1096)		
Variables	n	%	n	%	p
**Sex**					
Male	9853	95.96	415	4.04	**0.002**
Female	19,603	96.64	681	3.36	
**Age group**					
0–18^a^	5279	98.58	76	1.42	**<0.001**
19–45^b^	10,970	94.37	654	5.63	
46–65^c^	9316	97.32	257	2.68	
66–80^c^	3404	97.23	97	2.77	
≥81a,c	487	97.60	12	2.40	
**Department (state hospital)**					
Psychiatry	655	93.97	42	6.03	**<0.001**
Neurology	1270	95.85	55	4.15	
Infectious diseases	304	95.90	13	4.10	
Dermatology	1029	96.08	42	3.92	
Neurosurgery	594	96.59	21	3.41	
ENT	1375	96.90	44	3.10	
Pulmonology	1101	97.00	34	3.00	
General surgery	2034	97.23	58	2.77	
Obstetrics and gynecology	2931	97.38	79	2.62	
Orthopedics and trauma	2410	97.69	57	2.31	
Physical medicine and rehabilitation	1622	98.07	32	1.93	
Urology	1131	98.09	22	1.91	
Ophthalmology	1988	98.27	35	1.73	
Cardiology	1037	98.29	18	1.71	
Internal medicine	4806	98.81	58	1.19	
Pediatrics	3453	99.57	15	0.43	
Other departments	209	100.00	0	0.00	
**Date**					
1–9 February	10,833	96.61	380	3.39	0.256
10–19 February	9148	96.41	341	3.59	
20–29 February	9475	96.19	375	3.81	
**Time**					
Morning (09:00 AM–12:30 PM)	16,351	96.39	612	3.61	0.829
Afternoon (01:30 PM–05:00 PM)	13,105	96.44	484	3.56	

a,b,c : There is a statistically significant difference between the parameters shown with different letters.

**Table 4:** Factors associated with patient attendance at hospital appointments.

	State hospital				
	Attended (n = 27,949)		Did not attend (n = 625)		
Variables	n	%	n	%	p
**Sex**					
Male	9235	97.64	223	2.36	0.165
Female	18,714	97.90	402	2.10	
**Age group**					
0–18^a^	5083	99.05	49	0.95	**<0.001**
19–45^b^	10,136	96.72	344	3.28	
46–65^c^	8997	98.15	170	1.85	
66–80a,c	3267	98.37	54	1.63	
≥81a,b,c	466	98.31	8	1.69	
**Department**					
Psychiatry	655	93.97	42	6.03	**<0.001**
Neurology	1270	95.85	55	4.15	
Infectious diseases	304	95.90	13	4.10	
Dermatology	1029	96.08	42	3.92	
Neurosurgery	594	96.59	21	3.41	
ENT	1375	96.90	44	3.10	
Pulmonology	1101	97.00	34	3.00	
General surgery	2034	97.23	58	2.77	
Obstetrics and gynecology	2931	97.38	79	2.62	
Orthopedics and trauma	2410	97.69	57	2.31	
Physical medicine and rehabilitation	1622	98.07	32	1.93	
Urology	1131	98.09	22	1.91	
Ophthalmology	1988	98.27	35	1.73	
Cardiology	1037	98.29	18	1.71	
Internal medicine	4806	98.81	58	1.19	
Pediatrics	3453	99.57	15	0.43	
Other departments	209	100.00	0	0.00	
**Date**					
1–9 February^a^	9612	98.12	184	1.88	**0.005**
10–19 February^b^	8979	97.44	236	2.56	
20–29 February	9358	97.86	205	2.14	
**Time**					
Morning (09:00 AM–12:30 PM)	15,564	97.74	360	2.26	0.341
Afternoon (01:30 PM–05:00 PM)	12,385	97.91	265	2.09	

a, b, c : There is a statistically significant difference between the parameters shown with different letters.

**Table 5:** Factors associated with patient at dental center appointments.

	OHDC				
	Attended (n = 1507)		Did not attend (n = 471)		
**Variables**	**n**	**%**	**n**	**%**	**p**
**Sex**					
Male	618	76.30	192	23.70	0.925
Female	889	76.11	279	23.89	
**Age group**					
0–18^a^	196	87.89	27	12.11	**<0.001**
19–45^b^	834	72.9	310	27.1	
46–65^b^	319	78.57	87	21.43	
66–80^b^	137	76.11	43	23.89	
>81a,b	21	84.00	4	16.0	
**Date**					
1–9 February^a^	1221	86.17	196	13.83	**<0.001**
10–19 February^b^	169	61.68	105	38.32	
20–29 February^c^	117	40.77	170	59.23	
**Time**					
Morning (09:00 AM–12:30 PM)	787	75.75	252	24.25	0.627
Afternoon (01:30 PM–05:00 PM)	720	76.68	219	23.32	

a, b, c : There is a statistically significant difference between the parameters shown with different letters.
